# Desmopressin and bleeding risk in high-risk native kidney biopsy: updated meta-analysis of RCTs and observational studies

**DOI:** 10.1080/0886022X.2025.2549775

**Published:** 2025-08-31

**Authors:** Gabriel Sartori Pacini, Vanderlei Carlos Bertuol Junior, Renata Asnis Schuchmann, Arthur Gus Manfro, Gabriela Vieira Steckert, Cleuber Gea Martins FIlho, Roberto Ceratti Manfro, Andrea Carla Bauer

**Affiliations:** a Division of Nephrology, Hospital de Clínicas de Porto Alegre, Porto Alegre, Brazil; b Postgraduate Program in Medical Sciences, Endocrinology, Universidade Federal do Rio Grande do Sul, Porto Alegre, Brazil; c Division of Transplantation, Hospital de Clínicas de Porto Alegre, Porto Alegre, Brazil; d Postgraduate Program in Medicine, Medical Sciences, Universidade Federal do Rio Grande do Sul, Porto Alegre, Brazil; e Department of Internal Medicine, School of Medicine, Universidade Federal do Rio Grande do Sul, Porto Alegre, Brazil

**Keywords:** Desmopressin, kidney biopsy, bleeding, renal biopsy, DDAVP

## Abstract

**Background:**

Desmopressin (DDAVP) is sometimes used prophylactically to mitigate bleeding complications associated with kidney biopsy in patients with impaired kidney function, although its efficacy remains uncertain. We conducted a systematic review and meta-analysis to evaluate whether DDAVP reduces bleeding complications in high-risk patients undergoing native kidney biopsy.

**Methods:**

We searched PubMed, EMBASE, Cochrane, and ClinicalTrials.gov through April 2025 for randomized or observational studies comparing DDAVP to placebo or no intervention in adults with eGFR <60 mL/min/1.73 m^2^. The primary outcome was overall bleeding. Pooled risk ratios (RRs) were estimated using a random-effects model. Subgroup and sensitivity analyses, including leave-one-out diagnostics, were performed.

**Results:**

Nine studies (*n* = 2,470) were included. The pooled RR for bleeding was 0.61 (95% CI, 0.33–1.11), with substantial heterogeneity (*I*^2^ = 73.6%). Observational studies showed a significant reduction in bleeding (RR = 0.52; 95% CI, 0.44–0.61; *I*^2^ = 0%), whereas RCTs did not (RR = 0.87; 95% CI, 0.10–7.69; *I*^2^ = 89.3%). Sensitivity analysis identified one outlier; its exclusion reduced heterogeneity (*I*^2^ = 18.3%) and yielded a pooled effect (*p* < .0001). Safety outcomes were infrequently reported but appeared to be mild.

**Conclusions:**

While the overall pooled analysis did not reach statistical significance, results from observational studies and sensitivity analyses suggest a potential protective effect of DDAVP in reducing bleeding after kidney biopsy in high-risk patients. However, given the low certainty of evidence, these findings should be considered exploratory and hypothesis-generating. Larger, well-powered RCTs are warranted to confirm these findings and to better characterize safety.

## Graphical Abstract

**Figure:**
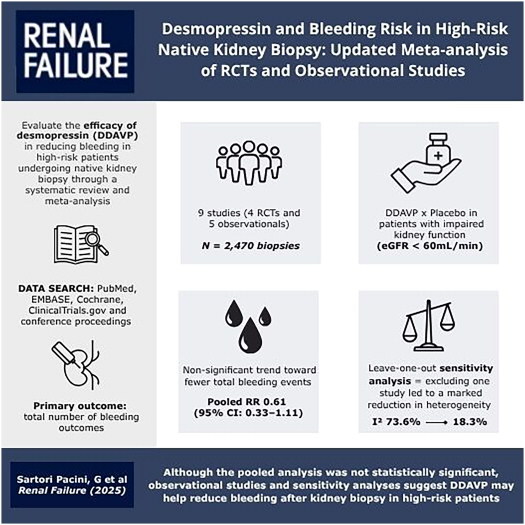

## 1. Introduction

Percutaneous kidney biopsy is a cornerstone in the diagnostic evaluation of kidney diseases [1]. This procedure is associated with an increased risk of bleeding complications, especially in patients with impaired kidney function, as uremia is known to cause platelet dysfunction and prolong bleeding time [1–3].

Desmopressin (DDAVP) has been shown to reduce bleeding complications in certain surgical procedures, but its role in percutaneous renal biopsy remains controversial [4]. Previous studies suggest that administration of DDAVP may decrease the risk of bleeding and hematoma size in patients undergoing percutaneous renal biopsy, with a favorable safety profile [5–7]. In a study by Manno et al. [5], a reduction in bleeding risk, particularly in hematoma formation, was observed in patients receiving DDAVP before the procedure. However, no significant difference was found for major bleeding events. On the other hand, some other studies reported similar bleeding complications in patients with and without DDAVP use, questioning the prophylactic benefit of DDAVP before renal biopsy in both high- and low-risk patients [8,9].

Currently, the use of DDAVP varies according to institutional protocols. However, the existing evidence, particularly for patients with impaired glomerular filtration rate (eGFR <60 mL/min), remains inconclusive. In this context, ‘high-risk’ refers to patients with reduced kidney function, defined as an estimated glomerular filtration rate (eGFR) <60 mL/min/1.73 m^2^, a threshold associated with uremic platelet dysfunction and increased bleeding risk. This systematic review and meta-analysis aimed to compare the use of DDAVP versus placebo or no treatment before native kidney biopsy in reducing the risk of bleeding in high-risk patients.

## 2. Materials and methods

### 2.1. Design and reporting

This systematic review with meta-analysis is registered in the PROSPERO database under the number CRD42024578572 (registered on 21 August 2024). It includes observational and randomized studies and follows the Preferred Reporting Items for Systematic Reviews and Meta-Analyses (PRISMA) guidelines [10].

### 2.2. Search strategy

We conducted a comprehensive literature search in PubMed-MEDLINE, EMBASE, Cochrane, ClinicalTrials.gov databases, and conference proceedings for studies published up to April 2025. The combination of MeSH terms and free-text words was: ‘kidney’ AND ‘biopsy’ AND ‘desmopressin’ NOT ‘mice’. The search terms are detailed in Supplementary Material (Appendix E1).

This systematic review and meta-analysis were conducted in accordance with the PRISMA 2020 guidelines, and the completed checklist is provided in the Supplementary Material (Appendix E3).

### 2.3. Inclusion and exclusion criteria

Studies were eligible for inclusion if they met the following criteria: (1) comparing DDAVP to placebo or no treatment; (2) patients undergoing native kidney biopsy; (3) renal impairment function (mean or median eGFR <60 mL/min/1.73 m^2^); and (4) similar eGFR between comparable groups. For this review, ‘high-risk’ was operationally defined as patients with impaired kidney function (eGFR <60 mL/min/1.73 m^2^), consistent with all included studies. Additional risk factors, such as platelet count or bleeding scores, were not uniformly reported and were therefore not used for stratification.

We excluded studies that (1) were not published in English; (2) were case reports, letters to the editor, or animal studies; (3) focused on kidney graft biopsies; (4) included patients without renal impairment; and (5) lacked sufficient data for analysis.

### 2.4. Study quality assessment

Two reviewers independently assessed the studies’ quality. The risk of bias was evaluated using the Cochrane Risk of Bias 2 (RoB-2) tool [11,12] in its five domains: randomization, deviations from the intended intervention, missing outcome data, outcome measurement, and selection of reported results.

For observational studies, the ROBINS-I tool was used to assess risk of bias across seven methodological domains, in accordance with Cochrane guidance [13]. Studies scoring 7–9 points were deemed high quality. Disagreements were resolved by consensus between the two reviewers.

### 2.5. Selection process, data extraction, and outcome measurements

Two reviewers independently screened titles and abstracts using Rayyan software [14]. Full texts were assessed for eligibility based on predefined inclusion and exclusion criteria. Discrepancies were resolved by consensus or by consulting a third reviewer.

Data extraction was also performed independently by two reviewers. It included the first author, year, study design, route of administration, intervention and control arms, number of patients, and baseline characteristics (age, sex, eGFR, blood pressure, hemoglobin, platelet count, and prothrombin time). The primary outcome was the incidence of total bleeding events. Secondary outcomes included major bleeding (defined as events requiring transfusion, intervention as embolization, nephrectomy, or resulting in mortality) and minor bleeding (defined as all other bleeding-related complications). Additional outcomes included hematoma, hematuria, need for transfusion or intervention, and reported adverse effects.

### 2.6. Statistical analyses

All statistical analyses were performed in R (version 2023.06.0 + 421) [15], using the meta and metaprop packages. Risk ratios (RRs) for binary outcomes were pooled using the metabin() function with a random-effects model based on the DerSimonian–Laird estimator, independent of *I*^2^ values, consistent with current Cochrane guidance. Heterogeneity was assessed through Cochran’s *Q* statistic, *I*^2^, and *τ*^2^. For outcomes involving prevalence, meta-analyses were conducted with metaprop(), applying logit transformation to stabilize variances. In instances of zero events in one arm, a continuity correction of 0.5 was implemented. Publication bias was explored using funnel plots and Egger’s regression test [16]. Pre-specified subgroup analyses were conducted by study design (RCTs vs. observational) and by the route of DDAVP administration (intravenous vs. intranasal). We assessed the certainty of evidence for the primary and key secondary outcomes using the Grading of Recommendations, Assessment, Development and Evaluation (GRADE) framework, as recommended by the Cochrane Handbook for Systematic Reviews of Interventions [12]. The results of this assessment are presented in a Summary of Findings table.

## 3. Results

### 3.1. Study selection and description

**Figure 1.**
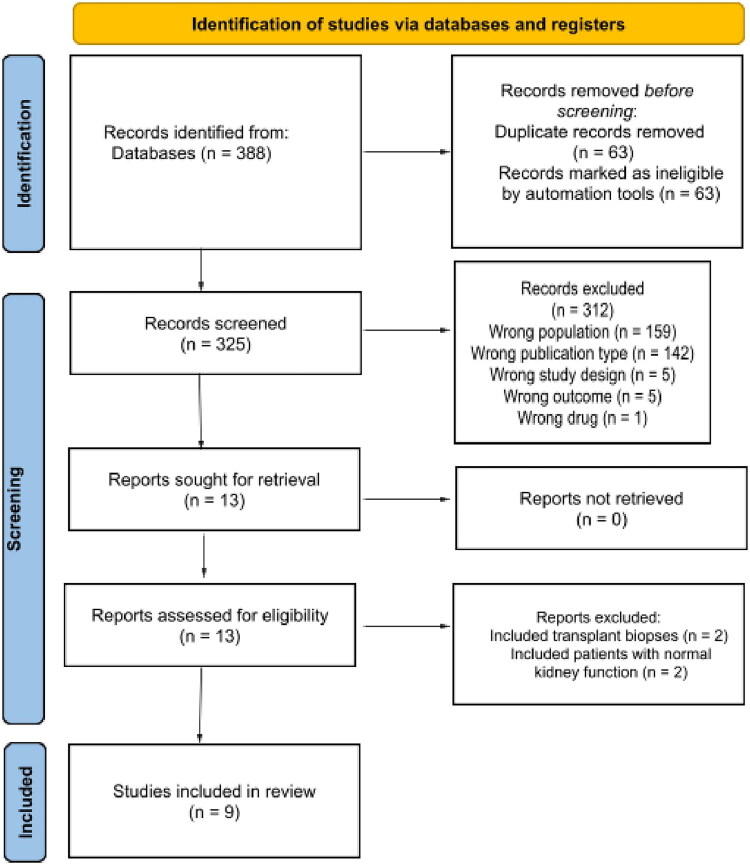
Study flow diagram for database search and study selection.

The initial search yielded 388 articles. After excluding 312 articles based on abstract reviews, 12 articles were selected for full-text review. Of these, nine studies met the eligibility criteria [6–8,17–22]. A detailed selection process is presented in Figure 1.

A total of 2,470 biopsies were included, of which 1,347 received DDAVP and 1,123 received normal saline or no treatment. The median number of biopsies was 200 (IQR 152–413). Among patients, the median number who received DDAVP was 101 (IQR 74–204), normal saline was 69 (IQR 55–84), and no treatment was 125 (IQR 105–156). DDAVP was administered via intranasal, subcutaneous, or intravenous routes, at doses of 3 µg/kg or fixed doses (150–160 µg). The characteristics of the included studies are summarized in Table 1.

**Table 1.** (A) Characteristics of randomized controlled trials (RCTs) and (B) characteristics of observational studies.

Author		Year	Country		Study design and setting	Inclusion criteria		*N*		Age (mean ± SD)					Male (%)	eGFR (mean ± SD)				Route, time and dose of DDAVP			Control
										Intervention			Control			Intervention		Control					
*(A) Characteristics of RCTs*																							
Chakrabarti et al. [19]	2025		India		RCT, double blind, unicenter		eGFR < 60 mL/min/1.73 m^2^		152		39.08 ± 15.03	38.83 ± 15.6			50.9		20.01 ± 13.14		22.08 ± 16.48		IN 3 mcg/kg 1 h before	IN Normal saline	
Sethi et al. [8]	2023		India		RCT, double blind, pilot study, unicenter		eGFR < 45 mL/min/1.73 m^2^		80		42.90 ± 14.35	45.25 ± 11.00			62.5		22.41 ± 12.37		19.23 ± 12.86		IN 150 mcg 1 h before	IN Normal saline	
Sattari et al. [18]	2022		Iran		RCT, double blind, unicenter		eGFR > 15, <90 mL/min/1.73 m^2^		120		46.85 ± 14.94	43.73 ± 16.88			48.3		54.14 ± 20.52		49.41 ± 5.63		IN 3 mcg/kg 1 h before	IN Normal saline	
Prasad et al. [22]	2025		India		RCT, double blind, unicenter		eGFR > 15, <90 mL/min/1.73 m^2^		203		42.64 ± 15.13	38.53 ± 15.10			62.56		31 (14.7–66.5)		30 (17.5–73)^a^		IN 300 mcg 1 h before	IN Normal saline	
*(B) Characteristics of observational studies*																							
Jose et al. [7]	2022			India	Retrospective, unicenter		eGFR < 30 mL/min/1.73 m^2^		432		39.2 ± 15.4	38.9 ± 15.5		63.6			10.89 ± 3.71		11.62 ± 4.22		IN 160 mcg/kg 1 h before	NR	
Leclerc et al. [21]	2020			Canada	Retrospective, unicenter		eGFR > 15, <90 mL/min/1.73m^2^		413		60 (44–70)^a^	64 (51–69)^a^		54.7			28 (15–51)^a^		45 (28–82)^a^		IV 0.3 mcg/kg	NR	
Rao and Chandra [6]	2020			India	Prospective and retrospective, unicenter		eGFR < 60 mL/min/1.72 m^2^		194		40.7 ± 16.8	36 ± 16.8		NR			36.58 ± 115.33		34.92 ± 14.58		IN 150 mcg 1 h before	NR	
Peters et al. [17]	2018			Sweden	Prospective and retrospective, multicenter		Cr > 150 μmol/L		576		55.6 ± 16.6	58.5 ± 17		70.5			22.3 ± 11		22.4 ± 11		SC 0.3 mcg/kg	NR	
Rogers and Barney [20]	2016			Netherlands	Retrospective, unicenter		eGFR < 30 mL/min/1.72 m^2^		200		NR			NR			NR				IV 0.3 mcg/kg	NR	

M: male; eGFR: estimated glomerular filtration rate; RCT: randomized controlled trial; SC: subcutaneous; IV: intravenous; IN: intranasal; NR: not reported; BP: blood pressure.

Data in each column refer first to patients in the control group, followed by those in the intervention group. Data are presented as mean ± SD.

^a^ Data presented as interquartile range (IQR).

### 3.2. Outcomes

**Figure 2.**
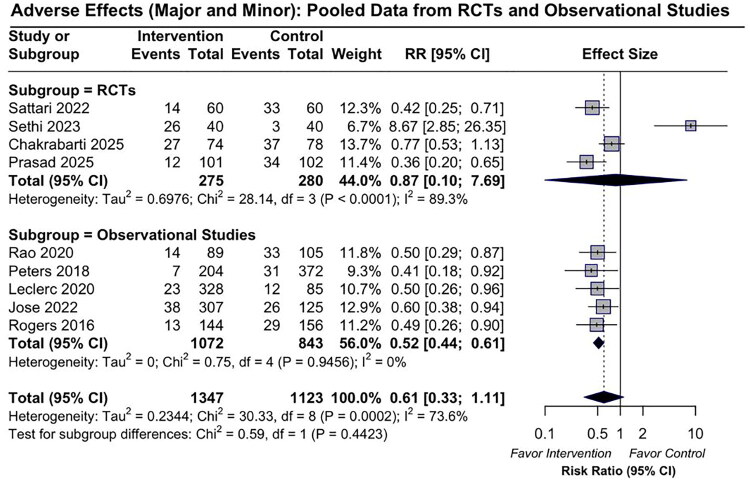
Forest plot of pooled risk ratios for bleeding events comparing DDAVP with placebo or no treatment in RCTs and observational studies. The *Q* statistic (Chi^2^) and *I*^2^ indicate the degree of between-study heterogeneity.

The updated pooled analysis of nine studies (2,470 patients) showed a non-significant trend toward fewer total bleeding events in the DDAVP group compared to the placebo/no treatment group (pooled RR 0.61 [95% CI: 0.33–1.11]), with considerable heterogeneity (*I*^2^ = 73.6%). In the subgroup analysis of randomized controlled trials (RCTs), the pooled RR was 0.87 [95% CI: 0.10–7.69] with substantial heterogeneity (*I*^2^ = 89.3%). Among observational studies, the pooled RR was 0.52 [95% CI: 0.44–0.61], with no heterogeneity (*I*^2^ = 0%) (Figure 2).

**Figure 3.**
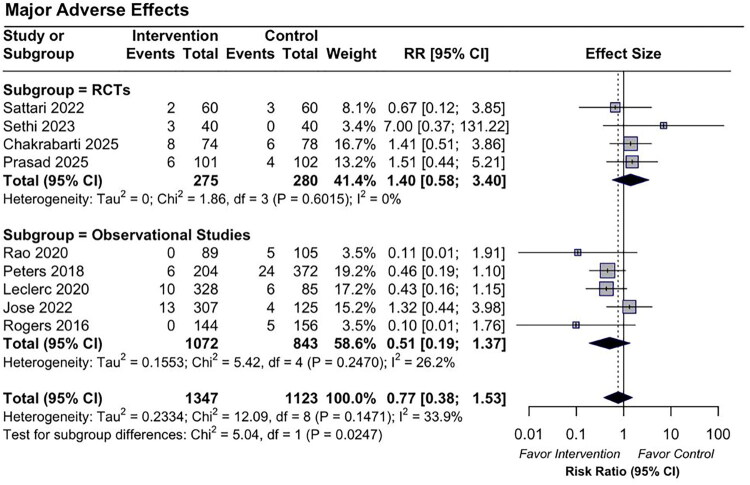
Forest plot of pooled risk ratios for major bleeding events comparing DDAVP with placebo or no treatment in randomized controlled trials (RCTs) and observational studies.

**Figure 4.**
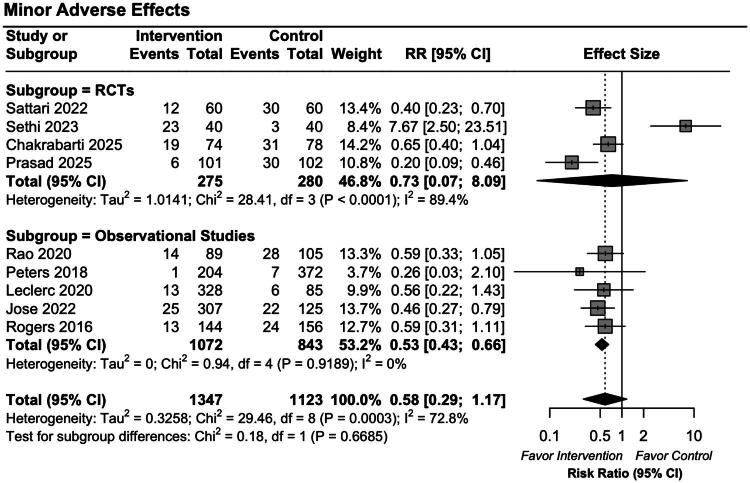
Forest plot of pooled risk ratios for minor bleeding events comparing DDAVP with placebo or no treatment in RCTs and observational studies.

For major bleeding events, no significant difference was observed between the groups (pooled RR 0.77 [95% CI: 0.38–1.53]). The RCT subgroup showed a pooled RR of 1.4 [95% CI: 0.58–3.4], while the observational studies reported a pooled RR of 0.51 [95% CI: 0.19–1.37], both with wide CIs crossing the null (Figure 3). For minor bleeding events, the overall pooled analysis also did not reveal a significant difference (pooled OR 0.58 [95% CI: 0.29–1.17]). In the RCT subgroup, the pooled RR was 0.73 [95% CI: 0.07–8.09]. A statistically significant reduction in minor bleeding events was found in the observational studies (pooled OR 0.53 [95% CI: 0.43–0.66]) (Figure 4). The certainty of evidence for these outcomes, assessed using the GRADE framework, was rated as low due to substantial heterogeneity and imprecision. A detailed Summary of Findings table is provided in Appendix E4.

No statistically significant differences were found in the subgroup analyses by administration route. The intranasal DDAVP showed no effect (pooled RR 0.69 [95% CI 0.23–2.04]; Figure S1). Other subgroup analyses could not be conducted due to the limited availability of relevant data.

### 3.3. Risk of bias and sensitivity analysis

**Figure 5.**
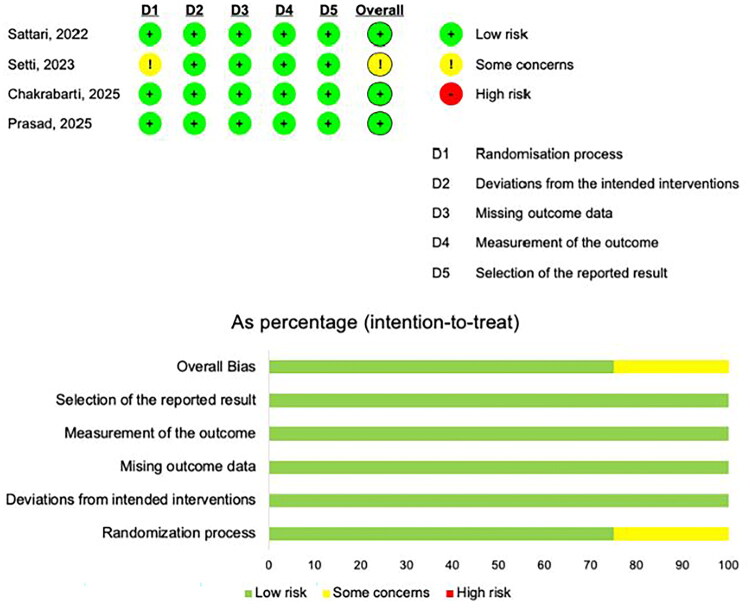
Risk of bias for RCTs.

Risk of bias in the observational studies was assessed using the ROBINS-I tool. Most studies were judged as having a moderate risk of bias, primarily due to potential residual confounding and incomplete reporting of adverse events (Figure S2). Among the four RCTs, three were rated as low risk of bias and one had some concerns, as evaluated using the RoB-2 tool (Figure 5).

**Figure 6.**
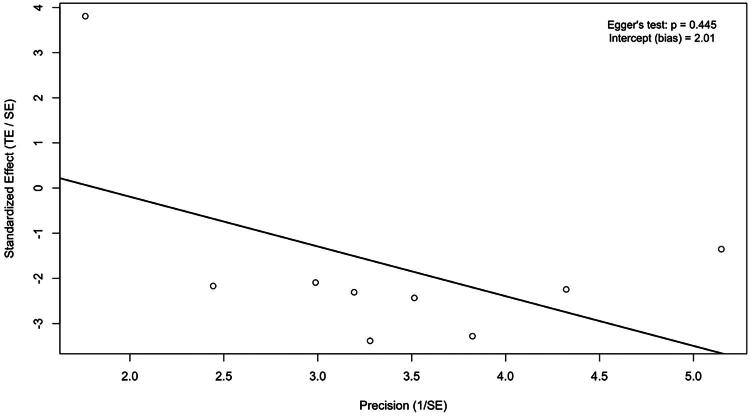
Egger’s regression plot showing standardized effect size (TE/SE) plotted against study precision (1/SE).

To explore heterogeneity and assess the robustness of the pooled effect estimates, a series of diagnostic and sensitivity analyses was conducted. The Baujat plot (Figure S3) and funnel plot (Figure S4) identified one study as an influential outlier, contributing disproportionately to both the effect size and heterogeneity. Although Egger’s regression test did not indicate significant publication bias (intercept = 2.01; *p* = .445) (Figure 6), the study’s leverage justified further investigation.

**Figure 7.**
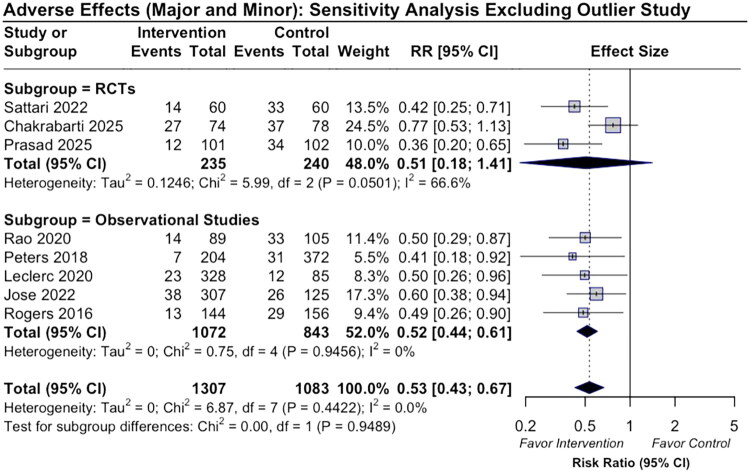
Forest plot of pooled risk ratios for bleeding events comparing DDAVP with placebo or no treatment in RCTs and observational studies, excluding the outlier study. The *Q* statistic (Chi^2^) and *I*^2^ indicate the degree of between-study heterogeneity.

Leave-one-out sensitivity analysis revealed that excluding this single study led to a marked reduction in heterogeneity (*I*^2^ from 92.6% to 18.3%; *τ*^2^ = 0.017; *Q* = 8.21) and yielded a pooled risk ratio (RR = 0.52 [95% CI: 0.38–0.71]; *p* < .0001) (Figure 7). No other individual study had a comparable impact on model estimates. Full results for all exclusions, including heterogeneity statistics (*I*^2^, *τ*^2^, and *Q*), are presented in Appendix E2.

**Figure 8.**
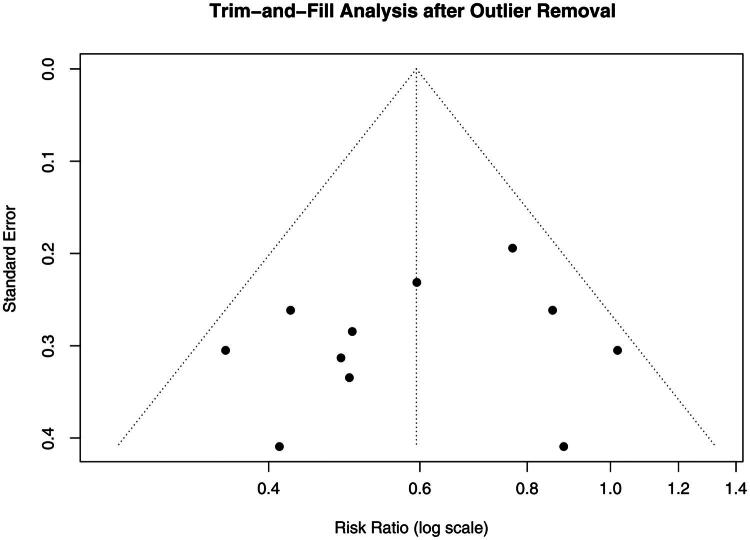
Trim-and-fill funnel plot.

Visual inspection of the funnel plot (Figure S4) revealed noticeable asymmetry, suggesting the possibility of publication bias. Although Egger’s regression test did not reach statistical significance, the visual pattern supported the application of the trim-and-fill method as a sensitivity analysis. As an additional step, a trim-and-fill procedure was applied after excluding the outlier [8]. The method imputed three hypothetical missing studies on the right side of the funnel plot (Figure 8). Importantly, this adjustment did not materially change the pooled effect estimate, further supporting the robustness of the results and the likely presence of minor publication bias.

## 4. Discussion

This updated meta-analysis evaluated the efficacy of DDAVP in reducing bleeding complications in patients with impaired renal function undergoing native kidney biopsy. The pooled analysis of nine studies involving 2,470 participants revealed a non-significant trend toward reduced bleeding events with DDAVP. However, deeper examination through subgroup and sensitivity analyses offers important insights into its potential benefit and the sources of heterogeneity.

Notably, observational studies showed a statistically significant reduction in bleeding events with DDAVP (RR = 0.52 [95% CI: 0.44–0.61]), and this result was consistent across analyses, with no observed heterogeneity (*I*^2^ = 0%). These findings suggest that DDAVP may offer a clinically meaningful reduction in bleeding in real-world populations. However, it is important to acknowledge that these findings may be subject to residual confounding and selection bias inherent to non-randomized designs. The stronger effect size in these studies, compared to the non-significant results in RCTs, suggests caution in inferring causality. The limited number and power of available RCTs underscore the urgent need for robust trials to validate these observations.

A critical component of this analysis was the identification of one of the studies [8] as an influential outlier. Sensitivity analysis showed that its exclusion led to a substantial improvement in model fit: heterogeneity dropped from *I*^2^ = 92.6% to 18.3%, and the pooled effect became evident (RR ≈ 0.52, *p* < .0001). No other individual study exerted a similar influence. While the methodology of the study was largely consistent with the other included trials, the divergent effect size may reflect random variation inherent to its small sample size (*n* = 80), as well as its pilot study nature. Pilot studies are typically smaller and designed to assess feasibility, safety, and preliminary efficacy, making their results more susceptible to random variation and less precise in estimating actual treatment effects. This finding underscores the need to interpret pooled estimates considering influential data points and reinforces the value of sensitivity diagnostics in meta-analyses.

An additional source of heterogeneity is the variability in the definition of bleeding complications across studies. Some studies defined bleeding as any hematoma or hematuria visible on imaging or clinically, while others limited their definition to events requiring intervention or transfusion. This heterogeneity in outcome definitions likely contributed to differences in reported effect sizes and complicates direct comparisons between studies.

While RCTs remain the gold standard for evaluating interventions, the available trials in this context were small, methodologically heterogeneous, and underpowered to detect differences in bleeding outcomes. Furthermore, the consistent signal observed in observational studies, combined with the robustness of the pooled estimate after exclusion of the outlier, suggests that DDAVP may confer a real clinical benefit in selected high-risk populations.

A previous systematic review found no superiority of DDAVP over placebo in preventing bleeding complications during percutaneous kidney biopsies [23]. It included only two articles, one of which suggested a potential benefit of DDAVP, but only in patients with normal renal function [5]. The physiological rationale for DDAVP use in patients with renal impairment is well-established. Uremic platelet dysfunction contributes to increased bleeding risk, and DDAVP enhances platelet adhesion by promoting the release of von Willebrand factor.

Major clinical guidelines, including those from KDIGO, the European Renal Association (ERA), and the American Society of Nephrology (ASN), do not currently recommend DDAVP as a routine prophylactic intervention for patients undergoing renal biopsy. Despite earlier studies showing shortened bleeding times in dialysis patients treated with DDAVP, most centers do not use it routinely [17]. Moreover, previous studies reporting potential benefits have not consistently demonstrated reductions in major or minor complications [5,17]. This variability reflects the inconsistent findings in the literature and highlights the need for further well-designed trials. A broader literature review underscores the heterogeneity of evidence and the absence of guideline-based consensus, reinforcing the need for carefully designed RCTs to clarify DDAVP’s role [24].

Regarding safety, DDAVP has been associated with an increased risk of hyponatremia compared to placebo [25]. In our meta-analysis, adverse events were infrequently reported [18–21] and, when described, were generally mild and reversible. This limits firm conclusions about safety. However, the low incidence of major complications and mortality in the included studies suggests an acceptable safety profile for single-dose DDAVP administration in this context. If confirmed by future adequately powered randomized trials, our findings may support updates to current guideline recommendations, particularly in selected high-risk populations.

This study has several limitations. The number of high-quality RCTs remains limited, and although the observational studies yielded consistent findings, they are inherently susceptible to residual confounding and selection bias. Furthermore, while Egger’s test did not indicate significant publication bias (*p* = .445), its low power in meta-analyses with fewer than 10 studies should be acknowledged. Therefore, the possibility of publication bias cannot be definitively ruled out. Moreover, a trim-and-fill analysis conducted after outlier removal imputed three hypothetical studies, suggesting possible minor publication bias, although the pooled effect size remained stable. This reinforces the robustness of our findings while acknowledging potential limitations in the published evidence base. Subgroup analyses by administration route did not reveal significant differences, and dose-dependent effects could not be assessed due to the small number of studies and heterogeneous dosing protocols. Moreover, a subgroup analysis focusing on patients requiring hemodialysis could not be performed, as most included studies did not report dialysis status or stratify bleeding outcomes by this variable. Given the increased bleeding risk in this population, future studies should report dialysis-specific data to allow targeted analyses.

Nevertheless, this is the most comprehensive and methodologically rigorous meta-analysis to date on DDAVP use in native kidney biopsy. The incorporation of sensitivity diagnostics and outlier detection strengthens the credibility of our findings. To support clinical interpretation of our findings and highlight areas for future investigation, we propose a simplified risk-stratification scheme for DDAVP use prior to kidney biopsy (Appendix E5).

In conclusion, while the overall analysis, including all studies, did not reach statistical significance, the evidence from observational studies and the results of sensitivity analyses suggest a potential protective effect of DDAVP in reducing bleeding complications during native kidney biopsy in high-risk patients. These results should be interpreted as exploratory and hypothesis-generating, rather than definitive. The identification and exclusion of a single outlier study revealed a statistically significant effect and substantially reduced heterogeneity, reinforcing the potential clinical relevance of DDAVP use. These findings justify its cautious application in selected cases and emphasize the urgent need for well-designed, adequately powered randomized trials to confirm its efficacy and guide standardized recommendations.

## Supplementary Material

Appendix E5.jpg

Appendix E3.docx

Appendix E4.docx

Appendix E1.docx

Appendix E2.xlsx

## References

[1] Yoshimoto M, Fujisawa S, Sudo M. Percutaneous renal biopsy well-visualized by orthogonal ultrasound application using linear scanning. Clin Nephrol. 1988;30(2):106–110. 3052950

[2] Brachemi S, Bollée G. Renal biopsy practice: what is the gold standard? World J Nephrol. 2014;3(4):287–294. doi: 10.5527/wjn.v3.i4.287. 25374824 PMC4220363

[3] Mannucci PM, Remuzzi G, Pusineri F, et al. Deamino-8-d-arginine vasopressin shortens bleeding time in uremia. N Engl J Med. 1983;308(1):8–12. doi: 10.1056/NEJM198301063080102. 6401193

[4] Crescenzi G, Landoni G, Biondi-Zoccai G, et al. Desmopressin reduces transfusion needs after surgery. Anesthesiology. 2008;109(6):1063–1076. doi: 10.1097/ALN.0b013e31818db18b. 19034103

[5] Manno C, Bonifati C, Torres DD, et al. Desmopressin acetate in percutaneous ultrasound-guided kidney biopsy: a randomized controlled trial. Am J Kidney Dis. 2011;57(6):850–855. doi: 10.1053/j.ajkd.2010.12.019. 21354681

[6] Rao NS, Chandra A. Intranasal desmopressin reduces renal biopsy-related bleeding and serum sodium levels in patients with reduced renal function. Clin Kidney J. 2020;13(6):1063–1067. doi: 10.1093/ckj/sfz114. 33391750 PMC7769509

[7] Jose L, Kaul A, Bhadauria D, et al. Desmopressin acetate before percutaneous ultrasound-guided kidney biopsy in patients with renal failure – is it really beneficial? Indian J Nephrol. 2022;32(5):430–434. doi: 10.4103/ijn.IJN_553_20. 36568600 PMC9775618

[8] Sethi J, Bansal S, Lal A, et al. Role of desmopressin acetate before percutaneous ultrasound-guided kidney biopsy in patients with kidney dysfunction. Indian J Nephrol. 2024;34(3):228–232. doi: 10.4103/ijn.ijn_34_23. 39114394 PMC11302129

[9] Athavale A, Kulkarni H, Arslan CD, et al. Desmopressin and bleeding risk after percutaneous kidney biopsy. BMC Nephrol. 2019;20(1):413. doi: 10.1186/s12882-019-1595-4. 31730448 PMC6858772

[10] Moher D, Liberati A, Tetzlaff J, et al. Preferred reporting items for systematic reviews and meta-analyses: the PRISMA statement. Int J Surg. 2010;8(5):336–341. doi: 10.1371/journal.pmed.1000097. 20171303

[11] Sterne JAC, Savović J, Page MJ, et al. RoB 2: a revised tool for assessing risk of bias in randomised trials. BMJ. 2019;366:l4898. doi: 10.1136/bmj.l4898. 31462531

[12] Higgins JPT, Thomas J, Chandler J, et al., editors. Cochrane handbook for systematic reviews of interventions version 6.3 [updated 2022 Feb]. Cochrane; 2022. Available from: www.training.cochrane.org/handbook

[13] Sterne JA, Hernán MA, Reeves BC, et al. ROBINS-I: a tool for assessing risk of bias in non-randomised studies of interventions. BMJ. 2016;355:i4919. doi: 10.1136/bmj.i4919. 27733354 PMC5062054

[14] Ouzzani M, Hammady H, Fedorowicz Z, et al. Rayyan—a web and mobile app for systematic reviews. Syst Rev. 2016;5(1):210. doi: 10.1186/s13643-016-0384-4. 27919275 PMC5139140

[15] Balduzzi S, Rücker G, Schwarzer G. How to perform a meta-analysis with R: a practical tutorial. Evid Based Ment Health. 2019;22(4):153–160. doi: 10.1136/ebmental-2019-300117. 31563865 PMC10231495

[16] Egger M, Smith GD, Schneider M, et al. Bias in meta-analysis detected by a simple, graphical test. BMJ. 1997;315(7109):629–634. doi: 10.1136/bmj.315.7109.629. 9310563 PMC2127453

[17] Peters B, Hadimeri H, Mölne J, et al. Desmopressin (Octostim^®^) before a native kidney biopsy can reduce the risk for biopsy complications in patients with impaired renal function: a pilot study. Nephrology. 2018;23(4):366–370. doi: 10.1111/nep.13004. 28107603

[18] Sattari SA, Shahoori A, Shahbazian H, et al. Desmopressin acetate in percutaneous ultrasound-guided native kidney biopsy in patients with reduced kidney function: a double-blind randomized controlled trial. Iran J Kidney Dis. 2022;16(4):238–245. doi: 10.52547/ijkd.6966. 35962638

[19] Chakrabarti U, Jhorawat R, Bajpai NK, et al. Effect of desmopressin in post kidney biopsy bleeding complication in patients with reduced renal function: a randomized controlled trial. Kidney360. doi: 10.34067/KID.0000000760. PMC12407119 40067357

[20] Rogers MJe, Barney EJ. Efficacy and cost-effectiveness of DDAVP administration prior to renal biopsy when eGFR is below 30: a retrospective chart review. J Am Soc Nephrol. 2016;364A:27.

[21] Leclerc S, Nadeau-Fredette AC, Elftouh N, et al. Use of desmopressin prior to kidney biopsy in patients with high bleeding risk. Kidney Int Rep. 2020;5(8):1180–1187. doi: 10.1016/j.ekir.2020.05.006. 32775817 PMC7403497

[22] Prasad N, Meyyappan J, Yadav D, et al. Randomized double-blind placebo-controlled trial of desmopressin for post-kidney biopsy bleeding. Kidney Int Rep. 2025;10(7):2436–2445. doi: 10.1016/j.ekir.2025.04.019. 40677359 PMC12266218

[23] Lim CC, Tan HZ, Tan CS, et al. Desmopressin acetate to prevent bleeding in percutaneous kidney biopsy: a systematic review. Intern Med J. 2021;51(4):571–579. doi: 10.1111/imj.14774. 32040251

[24] Bifari N, Alatawi Y, Abdel-Razaq WS, et al. The role of fixed-dose desmopressin in hemostatic outcomes of native and transplant kidney biopsies in a tertiary referral hospital. Healthcare. 2025;13(13):1553. doi: 10.3390/healthcare13131553. 40648578 PMC12249338

[25] Cheong M, Lee TY, Lee J, et al. No effect of desmopressin administration before kidney biopsy on the risk of major post-biopsy bleeding. Nefrologia. 2022;42(1):33–40. doi: 10.1016/j.nefroe.2020.12.008. 36153897

