# Supplementary figures and images for: Desmopressin and bleeding risk in high-risk native kidney biopsy: updated meta-analysis of RCTs and observational studies
Source: Ren Fail. 2025 Aug 31;47(1):2549775. doi: 10.1080/0886022X.2025.2549775 (PMC12404058; doi:10.1080/0886022X.2025.2549775)

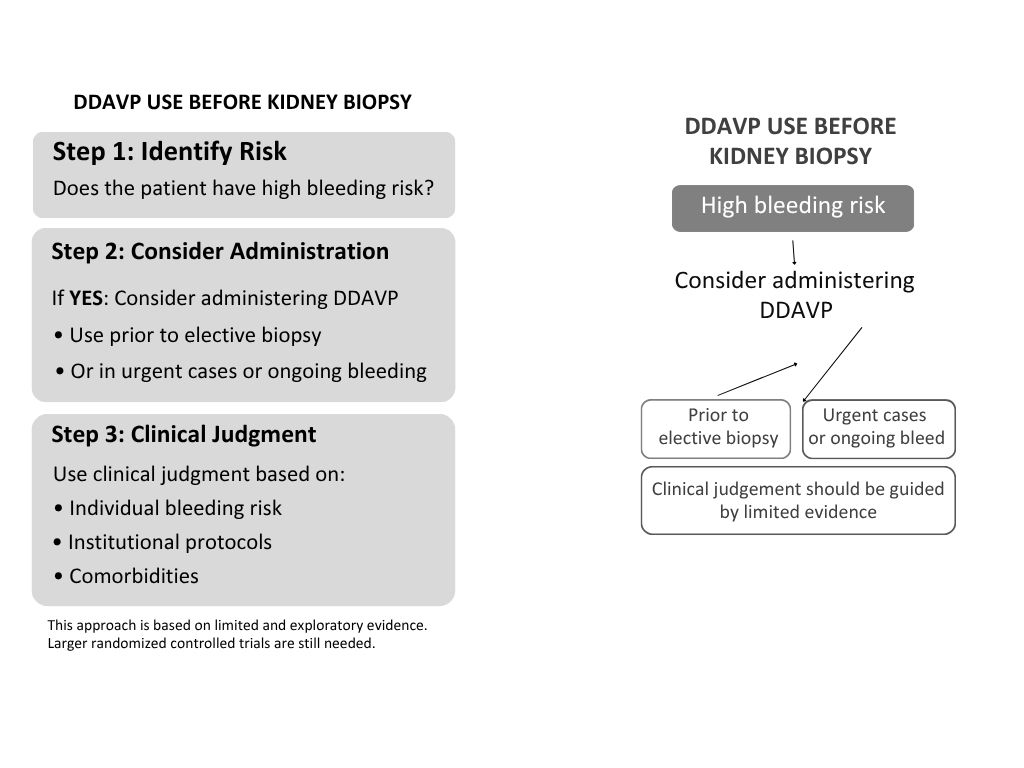

Supplement: Appendix E5.jpg [file IRNF_A_2549775_SM7868.jpg]
