# Supplementary material for: Desmopressin and bleeding risk in high-risk native kidney biopsy: updated meta-analysis of RCTs and observational studies
Source: Ren Fail. 2025 Aug 31;47(1):2549775. doi: 10.1080/0886022X.2025.2549775 (PMC12404058; doi:10.1080/0886022X.2025.2549775)
Supplement: Appendix E3.docx [file IRNF_A_2549775_SM7867.docx]

| Outcome | No. of Participants (Studies) | Relative Effect (95% CI) | Absolute Effect | Certainty of Evidence (GRADE) |
| --- | --- | --- | --- | --- |
| Total bleeding events | 2,470 (9 studies) | RR 0.57 (95% CI 0.21–1.52) | 116 per 1,000 fewer (from 92 more to 371 fewer) | ⬤⬤◯◯ Low |
| Major bleeding events | 2,470 (9 studies) | RR 0.69 (95% CI 0.32–1.51) | 42 per 1,000 fewer (from 25 more to 107 fewer) | ⬤⬤◯◯ Low |
| Minor bleeding events | 2,470 (9 studies) | RR 0.67 (95% CI 0.33–1.35) | 74 per 1,000 fewer (from 49 more to 196 fewer) | ⬤⬤◯◯ Low |
