# Supplementary material for: Desmopressin and bleeding risk in high-risk native kidney biopsy: updated meta-analysis of RCTs and observational studies
Source: Ren Fail. 2025 Aug 31;47(1):2549775. doi: 10.1080/0886022X.2025.2549775 (PMC12404058; doi:10.1080/0886022X.2025.2549775)
Supplement: Appendix E4.docx [file IRNF_A_2549775_SM7866.docx]

This table summarizes the results of a leave-one-out sensitivity analysis performed to evaluate the impact of each individual study on the pooled effect estimate. For each excluded study, the random-effects risk ratio (RR), 95% confidence interval (CI), and overall p-value are reported. The exclusion of Sethi et al., 2023, resulted in a statistically significant effect with markedly reduced heterogeneity, supporting the robustness of the overall findings.

| **Excluded Study** | **Pooled RR** | **95% CI** | **p-value** | **I² (%)** | **Tau²** | **Q** |
| --- | --- | --- | --- | --- | --- | --- |
| **Sattari 2022** | 0.66 | 0.38–1.15 | 0.140 | 75.6 | 0.270 | 28.73 |
| **Sethi 2023** | 0.39 | 0.32–0.49 | **0.000** | 0.0 | 0.0 | 6.87 |
| **Chakrabarti 2025** | 0.61 | 0.34–1.07 | 0.085 | 74.5 | 0.283 | 27.47 |
| **Prasad 2025** | 0.67 | 0.39–1.15 | 0.144 | 74.6 | 0.244 | 27.56 |
| **Rao 2020** | 0.65 | 0.37–1.13 | 0.123 | 76.7 | 0.279 | 30.05 |
| **Peters 2018** | 0.65 | 0.38–1.12 | 0.123 | 76.4 | 0.254 | 29.61 |
| **Leclerc 2020** | 0.64 | 0.37–1.12 | 0.118 | 76.8 | 0.269 | 30.11 |
| **Jose 2022** | 0.63 | 0.36–1.12 | 0.113 | 76.9 | 0.302 | 30.31 |
| **Rogers 2016** | 0.65 | 0.37–1.12 | 0.122 | 76.7 | 0.272 | 29.99 |
