# Supplementary material for: Desmopressin and bleeding risk in high-risk native kidney biopsy: updated meta-analysis of RCTs and observational studies
Source: Ren Fail. 2025 Aug 31;47(1):2549775. doi: 10.1080/0886022X.2025.2549775 (PMC12404058; doi:10.1080/0886022X.2025.2549775)
Supplement: Appendix E1.docx [file IRNF_A_2549775_SM7865.docx]

Appendix E1. Database Search Strategies

Database: Pubmed 1980 to Present

Search Date: 17/03/2025

Search Strategy:

#1 kidney [MeSH Terms]

#2 kidney

#3 #1 AND #2

#4 biopsy [MeSH Terms]

#5 biopsy

#6 #4 AND #5

#7 desmopressin [MeSH Terms]

#8 desmopressin

#9 #7 AND #8

#10 #3 AND #6 AND #9

#11 mice [MeSH Terms]

#12 mice

#13 #11 AND #12

#14 #10 NOT #13

***************************

03/2025 - 16

Database: EMBASE 1980 to Present

Search Date: 17/03/2025

Search Strategy:

#1 kidney biopsy

#2 desmopressin

#3 #1 AND #2

#4 mouse

#5 #3 NOT #4

**************************

03/2025 -

Database: Cochrane Central Register of Controlled Trials

Search Date: 17/03/2025

Search Strategy:

#1 MeSH descriptor: [kidney]

#2 MeSH descriptor: [biopsy]

#3 MeSH descriptor: [desmopressin]

#4 #1 AND #2 AND #3

#5 Mesh descriptor: [mice]

#6 #4 NOT #5

***************************

Database: Clinical Trials

Search date: 17/03/2025

Search Strategy: kidney biopsy and desmopressin

***************************
